# Serum heat shock protein 47 levels are elevated in acute interstitial pneumonia

**DOI:** 10.1186/1471-2466-14-48

**Published:** 2014-03-21

**Authors:** Tomoyuki Kakugawa, Shin-ichi Yokota, Yuji Ishimatsu, Tomayoshi Hayashi, Shota Nakashima, Shintaro Hara, Noriho Sakamoto, Hiroshi Kubota, Mariko Mine, Yasuhiro Matsuoka, Hiroshi Mukae, Kazuhiro Nagata, Shigeru Kohno

**Affiliations:** 1 Second Department of Internal Medicine, Nagasaki University School of Medicine, 1-7-1 Sakamoto, Nagasaki, Nagasaki 852-8501, Japan; 2 Department of Microbiology, Sapporo Medical University School of Medicine, Sapporo, Japan; 3 Department of Pathology, Nagasaki University Hospital, Nagasaki, Japan; 4 Department of Molecular and Cellular Biology, Institute for Frontier Medical Sciences, Kyoto University, Kyoto, Japan; 5 Department of Life Science, Faculty and Graduate School of Engineering and Resource Science, Akita University, Akita, Japan; 6 Biostatistics Section, Division of Scientific Data Registry, Atomic Bomb Disease Institute, Nagasaki University, Nagasaki, Japan; 7 Department of Respiratory Medicine, School of Medicine, University of Occupational and Environmental Health, Kitakyushu, Japan; 8 Laboratory of Molecular and Cellular Biology, Faculty of Life Sciences, Kyoto Sangyo University, Kyoto, Japan; 9 Present address: Research Laboratories, Research & Development Division, Kyowa Medex Co., Ltd, Shizuoka, Japan

**Keywords:** Heat shock protein 47, Acute interstitial pneumonia, Idiopathic interstitial pneumonia

## Abstract

**Background:**

Heat shock protein (HSP) 47, a collagen-specific molecular chaperone, is involved in the processing and/or secretion of procollagen. We hypothesized that HSP47 could be a useful marker for fibrotic lung disease. The aim of this study was to evaluate serum levels of HSP47 in patients with various idiopathic interstitial pneumonias (IIPs).

**Methods:**

Subjects comprised 9 patients with acute interstitial pneumonia (AIP), 12 with cryptogenic organizing pneumonia (COP), 16 with nonspecific interstitial pneumonia (NSIP), 19 with idiopathic pulmonary fibrosis (IPF), and 19 healthy adult volunteers.

**Results:**

Patients with AIP had serum HSP47 levels that were significantly higher than those of COP, NSIP or IPF patients and those of healthy volunteers. In contrast, serum levels of HSP47 among patients with COP, NSIP, IPF, and healthy volunteers did not differ significantly. Receiver operating characteristic curves revealed that the cut-off level for HSP47 that resulted in the highest diagnostic accuracy for discriminating between AIP and COP, NSIP, IPF, and healthy controls was 859.3 pg/mL. The sensitivity, specificity, and diagnostic accuracy were 100.0%, 98.5%, and 98.7%, respectively.

**Conclusion:**

The present results demonstrate that, among patients with various IIPs, serum levels of HSP47 were elevated specifically in patients with AIP.

## Background

Clinical courses of the various types of idiopathic interstitial pneumonias (IIPs) vary widely [1,2]. Although analysis of a surgical lung biopsy has traditionally been the gold standard for the pathological diagnosis of IIPs and is clinically relevant for selecting appropriate treatment [1,3], it involves performance of a relatively invasive procedure, especially for patients with advanced IIP. Accordingly, identifying circulating markers effective for evaluating and monitoring disease activity and distinguishing the various types would improve management of IIPs. A number of serum markers suggestive of interstitial lung disease have been reported, including surfactant protein (SP)-A, SP-D, and Krebs von den Lungen-6 (KL-6), a circulating, high molecular weight glycoprotein expressed by type II pneumocytes [4-13]. However, the clinical usefulness of these serum markers for distinguishing the several types of IIPs remains unclear.

Heat shock protein (HSP) 47 is a collagen-binding, stress-inducible protein localized to the endoplasmic reticulum. HSP47 serves as a collagen-specific molecular chaperone in intracellular processing during procollagen production [14-16]. Recent studies have demonstrated that HSP47 expression is highly tissue-and cell-specific—mainly restricted to phenotypically altered collagen-producing cells [17]. HSP47 expression is upregulated in animals with experimentally induced pulmonary fibrosis [18-20]. Previous studies have also shown that expression of human HSP47 is increased in fibrotic lesions of idiopathic pulmonary fibrosis (IPF), idiopathic nonspecific interstitial pneumonia (NSIP), and diffuse alveolar damage (DAD) [21-23]. Similar to the fibrotic lung diseases mentioned above, induction of HSP47 is consistently observed in other fibrotic diseases such as those affecting the liver, kidney, heart, eyes and skin [17,24-26].

We recently showed in an experimental pulmonary fibrosis model that collagen accumulation and disease progression were associated with the level of HSP47 protein expression [19]. In addition, HSP47 expression is higher in the lungs of patients with idiopathic usual interstitial pneumonia (UIP) than in those with collagen vascular disease-associated UIP and idiopathic NSIP [22]; idiopathic fibrotic NSIP patients with higher HSP47 expression in their lungs had poorer prognosis than patients with lower HSP47 expression [27]. These findings suggest that the expression of HSP47 in fibrotic lung tissue correlates with fibrotic disease activity.

We hypothesized that HSP47 may leak into the peripheral blood, and that it could be a useful marker for fibrotic lung disease. Although Yokota (one of the authors of the present manuscript) et al. previously reported that the serum levels of HSP47 did not differ significantly between patients with IPF and healthy controls [28], we recently reported that HSP47 serum levels in patients with acute exacerbation of IPF were found to be markedly higher than in patients with stable IPF [23]. These findings suggest that serum HSP47 could be a useful marker for IIPs. However, the precise association between serum levels of HSP47 and IIPs remains obscure. The purpose of this study was to evaluate serum HSP47 levels in patients with various IIPs.

## Methods

### Study population

The present study was a retrospective case–control study. Study subjects consisted of 56 patients who were admitted to Nagasaki University Hospital from April 1996 to March 2011, and 19 healthy adult volunteers. Patients included 9 with acute interstitial pneumonia (AIP), 12 with cryptogenic organizing pneumonia (COP), 16 with NSIP, and 19 with IPF. Diagnoses were made according to the official ATS/ERS/JRS/ALAT statement [2] and the American Thoracic Society/European Respiratory Society consensus criteria [1]. Patients had no signs or positive serological (or other) markers of collagen vascular disease. All patients diagnosed with AIP met the following criteria: 1) development or unexplained worsening of dyspnea within 30 days; 2) high-resolution computed tomography chest scans with new bilateral ground-glass opacities and/or consolidation; 3) PaO_2_/fraction of inspired oxygen (FiO_2_) ratio (P/F ratio) < 300 mmHg; and 4) absence of apparent infection, pneumothorax, pulmonary thromboembolism, heart failure or alternative causes of acute lung injury, such as trauma, blood infusion or toxic inhalation. Serological and urinary studies were performed, and were negative in all patients diagnosed with AIP, for the following pathogens and pathogen components: endotoxin, *Mycoplasma pneumoniae, Chlamydophila pneumoniae*, *Chlamydophila psittaci,* cytomegalovirus antigen, β-D glucan, *Legionella spp.* and *Streptococcus pneumoniae*. Blood, sputum, and urine cultures were also negative. Echocardiography demonstrated no evidence of heart failure in any of the patients. All NSIP and IPF diagnoses were confirmed pathologically in multiple lobes by open lung biopsy or video-assisted thoracoscopic surgery. Sera were obtained from patients at the time of diagnosis. Patient characteristics were collected from the clinical notes recorded at the time of diagnosis and included age, sex, P/F ratio, and alveolar-arterial difference of oxygen (A-a DO_2_). Serum concentrations of KL-6, SP-A, SP-D and lactate dehydrogenase (LDH) were also collected from the clinical notes recorded at the time of diagnosis. For records lacking data for these markers, measurements were done using preserved serum samples. The 30-and 90-day mortality rates were determined for all disease groups. In addition, sera were obtained from healthy volunteers to serve as control subjects, all of whom had normal chest radiographs, were free of symptoms and were not taking any medications.

The study protocol was approved by the Institutional Review Board of Nagasaki University Hospital and the Ethics Committee, Nagasaki University Graduate School of Biomedical Sciences. Written informed consent was obtained from all subjects.

### Sandwich enzyme-linked immunosorbent assay (ELISA) for determining HSP47 concentration

Sandwich ELISA for determining HSP47 concentration was carried out as described previously [28].

### Measurement of serum KL-6, SP-A, SP-D, and LDH levels

Serum levels of KL-6, SP-A, SP-D, and LDH were measured using specific kits according to the manufacturers’ protocols. KL-6 concentrations were measured using a sandwich-type electrochemiluminescence immunoassay kit (Picolumi KL-6, Sanko Junyaku Co., Tokyo, Japan). SP-A and SP-D levels were measured using sandwich-type enzyme immunoassay kits (SP-A test-F, Kokusai Shiyaku Co., Hyogo, Japan; and SP-D kit, Yamasa, Yamasa Shoyu Co., Tokyo, Japan). LDH levels were measured using an ultraviolet method with an L-type WAKO LDH kit (Wako Pure Chemical Industries, Ltd., Osaka, Japan). All assays were performed in duplicate. Data regarding these markers was not obtained from all enrolled patients because some preserved serum samples were not of sufficient volume. Data for the patients in whom these markers were measured (including their numbers) are presented in Results section.

### Immunohistochemistry

Immunohistochemistry was performed as described previously [22].

### Statistical analysis

Values for continuous variables are expressed as median (range). Differences among groups were determined by analysis of variance or the Kruskal-Wallis test for continuous variables and the χ^2^ test for categorical variables, as appropriate. If a significant difference was found by analysis of variance, pair-wise comparison was performed using the Scheffe method. The upper left corner coordinate point of the receiver operating characteristic curve was used to determine the optimum cutoff level for discriminating between AIP and COP, NSIP, IPF, and healthy volunteers. Statistical analysis was performed using a statistical software package (SAS 9.1.3, SAS Institute, Cary, NC, USA). P values <0.05 were considered statistically significant.

## Results

### Patient characteristics

Table 1 lists characteristics of enrolled patients. The P/F ratios of the AIP groups were significantly lower, and the A-a DO_2_ significantly higher, as compared with those of the COP, NSIP, and IPF groups.

**Table 1:** Patient characteristics

	**Healthy volunteer (N = 19)**		**COP (N = 12)**		**NSIP (N = 16)**		**IPF (N = 19)**		**AIP (N = 9)**		**p value**
Age (years)	35*	(27-59)	65.5	(38-87)	57.0	(28-74)	64.0	(34-75)	75.0	(53-81)	<0.001
Sex (male/female)	11/8		7/5		6/10		13/6		6/3		N.S.
Smoking (s/ex/n)	0/0/19		2/4/6		3/5/8		6/5/8		5/1/3		0.002
P/F ratio (mmHg)	-		385.0	(289.5-490.5)	399.3	(343.3-442.4)	389.8	(283.3-491.9)	102.3^¶^	(59.9-254.8)	<0.001
A-a DO_2_ (mmHg)	-		19.9	(-5.14-46.9)	15.3	(2.6-37.9)	17.1	(-3.5-52.7)	402^¶^	(58.6-611.4)	<0.001

Data presented as median (range).

N = number of patients; COP = cryptogenic organizing pneumonia; NSIP = nonspecific interstitial pneumonia; IPF = idiopathic pulmonary fibrosis; AIP = acute interstitial pneumonia; N.S. = not significant; s/ex/n = current smoker/ex-smoker/nonsmoker; P/F ratio = PaO_2_/fraction of inspired oxygen (FiO_2_) ratio; A-a DO_2_ = alveolar-arterial difference of oxygen.

*P < 0.01 compared with COP, NSIP, IPF, and AIP.

^¶^P < 0.01 compared with COP, NSIP, and IPF.

### Disease outcomes

In the AIP group, 30-day mortality was 44.4% (4 of 9 patients), and 90-day mortality was 66.7% (6 of 9 patients). In contrast, none of the patients in the COP, NSIP, or IPF groups died within 90 days.

### Serum levels of HSP47, KL-6, SP-A, SP-D and LDH

**Figure 1:**
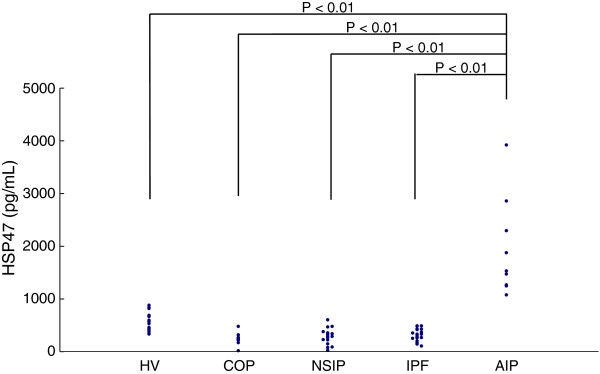
Scattergram of serum heat shock protein (HSP) 47 in patients with cryptogenic organizing pneumonia (COP), nonspecific interstitial pneumonia (NSIP), idiopathic pulmonary fibrosis (IPF), acute interstitial pneumonia (AIP), and in healthy volunteers (HV).

Serological data are presented in Table 2 and Figure 1. Serum levels of HSP47 in patients with AIP were significantly higher than in those with COP, NSIP, IPF, or in healthy volunteers. Serum levels of HSP47 among patients with COP, NSIP, IPF, and healthy volunteers were not significantly different (Figure 1). Serum levels of KL-6 in patients with NSIP and IPF were significantly higher than in those with COP and in healthy volunteers. Serum levels of SP-A in patients with IPF and AIP were significantly higher compared to those in healthy volunteers. Serum levels of SP-A in patients with IPF were significantly higher than in those with COP and NSIP. Serum levels of SP-D in patients with AIP were significantly higher compared to those in healthy volunteers. Serum levels of LDH in patients with AIP were significantly higher than in those with COP, NSIP, IPF, or in healthy volunteers. Serum levels of LDH in patients with NSIP and IPF were significantly higher compared to those in healthy volunteers.

**Table 2:** Serum concentrations of HSP47, KL-6, SP-A, SP-D, and LDH

	**Healthy volunteer (N = 19)**		**[n]**	**COP (N = 12)**		**[n]**	**NSIP (N = 16)**		**[n]**	**IPF (N = 19)**		**[n]**	**AIP (N = 9)**		**[n]**	**p value**
HSP47 (pg/mL)	565.8	(332.1-879.8)	[19]	239.1	(16.6-476.6)	[12]	290.7	(24.8-603.0)	[16]	330.9	(105.1-487.6)	[19]	1530.2*	(1075.1-3919.9)	[9]	<0.001
KL-6 (U/mL)	193.0	(144-322)	[19]	427.5	(172-1310)	[10]	1568.5^¶^	(192-4745)	[14]	1460^¶^	(444-4340)	[15]	332.5	(201-2200)	[8]	<0.001
SP-A (ng/mL)	22.7	(12.1-60.8)	[19]	52.8	(20.6-129)	[8]	48.9	(20.3-127)	[12]	103^§^	(62.4-355)	[15]	138^£^	(43.8-148)	[6]	<0.001
SP-D (ng/mL)	17.3	(17.3-58.6)	[19]	105.7	(27.8-247)	[8]	477.0	(17.2-942)	[13]	316.0	(93.1-721)	[14]	417^#^	(72.1-4510)	[8]	<0.001
LDH (IU/L)	124.5	(20-246)	[19]	164.0	(132-236)	[12]	212.5^#^	(135-738)	[14]	233^#^	(113-416)	[15]	380*	(231-736)	[9]	<0.001

Data presented as median (range).

N = number of patients; n = number of patients examined; COP = cryptogenic organizing pneumonia; NSIP = nonspecific interstitial pneumonia; IPF = idiopathic pulmonary fibrosis; AIP = acute interstitial pneumonia; HSP47 = heat shock protein 47; KL-6 = Krebs von den Lungen-6; SP-A = surfactant protein-A; SP-D = surfactant protein-D; LDH = lactate dehydrogenase.

*P < 0.01 compared with COP, NSIP, IPF, and healthy volunteers.

^¶^P < 0.01 compared with healthy volunteers; P < 0.05 compared with COP.

^§^P < 0.01 compared with NSIP and healthy volunteers; P < 0.05 compared with COP.

^£^P < 0.01 compared with healthy volunteers.

^#^P < 0.05 compared with healthy volunteers.

### Receiver operating characteristic curve

**Figure 2:**
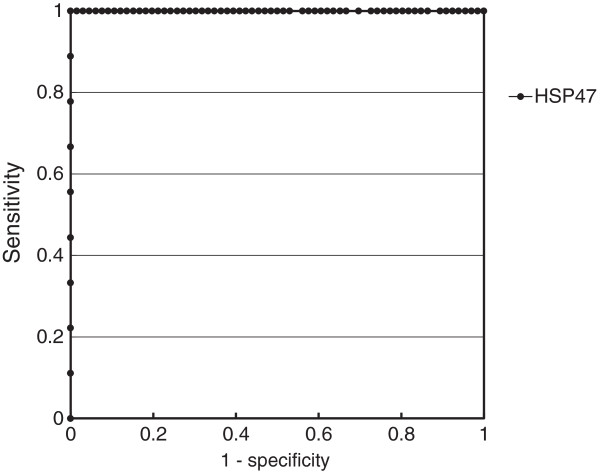
**Receiver operating characteristic curve for serum levels of heat shock protein (HSP) 47 in patients with various interstitial pneumonias.** Highest diagnostic accuracy cut-off level for HSP47 was 859.3 pg/mL, which discriminated between AIP and the other IIPs studied and healthy volunteers with 100% sensitivity, 98.5% specificity, and a diagnostic accuracy of 98.7%. Use of serum HSP47 level for diagnosis of AIP resulted in the area under the curve of 1.000.

Based on a receiver operating characteristic curve (Figure 2), the cut-off level of HSP47 that resulted in the highest diagnostic accuracy for discriminating between AIP and COP, NSIP, IPF, and healthy volunteers was 859.3 pg/mL. This value discriminated between AIP and COP, NSIP, IPF, and healthy volunteers with 100% sensitivity and 98.5% specificity. The diagnostic accuracy was 98.7%. Use of serum HSP47 levels for diagnosis of AIP resulted in an area under the curve of 1.000.

### Histopathological and immunohistochemical findings

**Figure 3:**
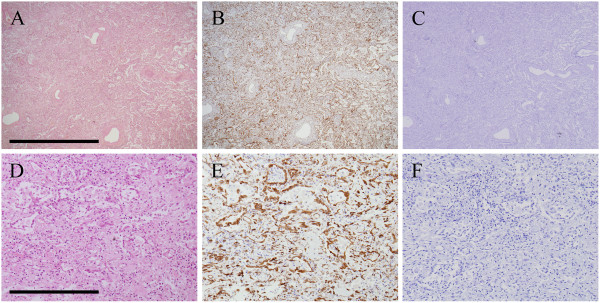
**Photomicrographs of histological and immunohistochemical studies of representative autopsy specimens with diffuse alveolar damage. A**-**B** and **D**-**E** are sequentially sectioned pairs. **A**, **D**; hematoxylin-eosin staining. **B**, **E**; HSP47 expression. **C**, **F**; Negative control studies using non-specific immunoglobulin-G. Scale bars: A-C = 2 mm, D-F = 400 μm.

Photomicrographs of histological and immunohistochemical studies of representative DAD autopsy specimens are shown in Figure 3. This DAD patient was given a final diagnosis of AIP. Figure 3 A-B and D-E depict pairs of sequential sections. At low magnification, diffuse involvement, including interstitial edema and inflammation, was seen (Figure 3 A-B). The expression of HSP47 in DAD was diffuse and higher than in UIP surgical lung biopsy specimens (data not shown). Histopathological examination at high magnification revealed interstitial edema, fibrosis, and inflammation in the DAD tissue. The expression of HSP47 was noted predominantly in fibroblasts, epithelial cells, and endothelial cells in DAD (Figure 3 D-E). Negative control studies using non-specific immunoglobulin-G revealed no positive cells (Figure 3 C, F).

## Discussion

In the present study, HSP47 serum levels in patients with AIP were found to be markedly higher than in patients with COP, NSIP, IPF, or in healthy volunteers. To the best of our knowledge, this is the first study to evaluate HSP47 serum levels of patients with AIP.

Despite extensive research conducted in the past, the precise molecular mechanisms leading to the lung injury and fibrosis of AIP are poorly understood. Previous reports have suggested that KL-6, SP-A, and SP-D might be useful markers for interstitial pneumonias [4-13,29]. It was assumed that serum levels of these markers in AIP patients would be higher than those in COP, NSIP and IPF patients: however, the current study demonstrated that they did not significantly differ from those in patients with other IIPs. In contrast, serum HSP47 levels were markedly elevated in AIP, but not in COP, NSIP, and IPF, indicating that the molecular mechanisms generating pathogenic fibrosis affecting serum HSP47 levels may differ distinctly between AIP and other IIPs.

Previous studies demonstrated that HSP47 expression in lung was higher in patients with UIP than in those with COP and NSIP, and in controls [22,30]. In contrast, the present study indicated that HSP47 levels in serum were elevated only in patients with AIP. Although a precise mechanism for the above unexpected observation was not elucidated in this study, the following is hypothesized: 1) We recently reported that HSP47 expression was higher in DAD lung that resulted in a final diagnosis of acute exacerbation of IPF than in UIP lungs, and that HSP47 serum levels in patients with acute exacerbation of IPF were found to be markedly higher than in patients with stable IPF [23]. The present study also demonstrated that HSP47 expression in lung was markedly high in a DAD patient who was given a final diagnosis of AIP. The amount of HSP47 in the lung, which has been reported to correlate with fibrotic disease activity [19,22,27,31], might correlate with serum levels. 2) Elevation of serum HSP47 in AIP might be due to distinctive characteristics of DAD, including severe inflammation, tissue destruction, alveolar epithelial and endothelial injury with increased vascular permeability [32]. These changes and high expression of HSP47 in epithelial cells and endothelial cells may possibly induce leakage of HSP47 protein into the extracellular matrix.

We also compared serum levels of HSP47 in the patients with acute exacerbation of IPF, which were reported in our previous study [23], with those of the AIP patients in the present study. Serum levels of HSP47 in patients with AIP did not differ significantly from those in patients with acute exacerbation of IPF (data not shown). Considering data from the present study together with our previous study [23] led us to the conclusion that serum HSP47 might be a useful marker to identify patients with DAD. Although patients with AIP and acute exacerbation of IPF histologically manifest as DAD, the histopathologic pattern of DAD is also seen in other types of lung injury. Hence, the serum HSP47 level might be a useful marker for other fatal and rapidly progressive fibrotic lung diseases which histologically manifest as DAD, regardless of etiology. Further investigations regarding the precise mechanisms involved are needed.

Some limitations to this study should be noted. First, the number of patients enrolled was small. Second, due to the small study population, the present study did not reveal whether serum HSP47 levels were correlated with disease severity or mortality. Third, it is not yet clear whether evaluation of sequential changes in HSP47 serum levels is useful to monitor disease progression and response to treatment. Fourth, it would be meaningful to evaluate serum levels of HSP47 in patients with acute respiratory distress syndrome (ARDS). According to our hypothesis, serum HSP47 levels might be elevated in patients with ARDS. A prospective multicenter study with a larger patient cohort, including ARDS patients, is planned in order to overcome the above-mentioned limitations of the present study.

## Conclusions

In conclusion, this study demonstrated that serum HSP47 levels were elevated in patients with AIP. This finding suggests that the underlying fibrogenic mechanisms affecting HSP47 levels might differ between AIP and other IIP patients. Further studies involving larger patient cohorts are warranted to determine whether serum HSP47 is a useful disease marker of AIP.

## Abbreviations

IIPs: Idiopathic interstitial pneumonias; SP: Surfactant protein; KL-6: Krebs von den Lungen-6; HSP: Heat shock protein; IPF: Idiopathic pulmonary fibrosis; NSIP: Nonspecific interstitial pneumonia; DAD: Diffuse alveolar damage; UIP: Usual interstitial pneumonia; AIP: Acute interstitial pneumonia; COP: Cryptogenic organizing pneumonia; P/F ratio: PaO_2_/fraction of inspired oxygen ratio; A-a DO2: Alveolar-arterial difference of oxygen; LDH: Lactate dehydrogenase; ELISA: Enzyme-linked immunosorbent assay; ARDS: Acute respiratory distress syndrome.

## Competing interests

T. Kakugawa received a research grant from Takeda Science Foundation and the Kato Memorial Trust for Nambyo Research. T. Kakugawa and S. Yokota have a patent application pending for research related to this manuscript.

## Authors’ contributions

TK made substantial contributions to the study conception and design. TK, NS, SH, SN and YI were involved in collecting clinical samples. SY made a substantial contribution to the determination of serum levels of HSP47 by ELISA. HK and YM made substantial contributions to the preparation of recombinant HSP47 protein. TK made a substantial contribution to the immunohistochemistry. TK and TH made pathological assessments. TK and MM were involved in statistical analysis. TK was involved in drafting the article. SY, YI, NS, HM, KN and SK were involved in revising the article critically for important intellectual content. All authors read and approved the final manuscript.

## Pre-publication history

The pre-publication history for this paper can be accessed here:

http://www.biomedcentral.com/1471-2466/14/48/prepub
